# The mushroom *Ganoderma lucidum* suppresses breast-to-lung cancer metastasis through the inhibition of pro-invasive genes

**DOI:** 10.3892/ijo.2014.2375

**Published:** 2014-04-09

**Authors:** JAGADISH LOGANATHAN, JIAHUA JIANG, AMANDA SMITH, ANDREJ JEDINAK, ANITA THYAGARAJAN-SAHU, GEORGE E. SANDUSKY, HARIKRISHNA NAKSHATRI, DANIEL SLIVA

**Affiliations:** 1 Cancer Research Laboratory, Methodist Research Institute, Indiana University Health, Indianapolis, IN 46202;; 2 Departments of Pathology, Indiana University School of Medicine, Indianapolis, IN 46202, USA; 3 Biochemistry and Molecular Biology, Indiana University School of Medicine, Indianapolis, IN 46202, USA; 4 Surgery, Indiana University School of Medicine, Indianapolis, IN 46202, USA; 5 Medicine, Indiana University School of Medicine, Indianapolis, IN 46202, USA; 6 Indiana University Simon Cancer Center, Indiana University School of Medicine, Indianapolis, IN 46202, USA

**Keywords:** mushroom, *Ganoderma lucidum*, polysaccharides, triterpenes, breast-to-lung cancer metastases, gene expression, cell migration

## Abstract

Breast cancer metastasis is one of the major reasons for the high morbidity and mortality of breast cancer patients. In spite of surgical interventions, chemotherapy, radiation therapy and targeted therapy, some patients are considering alternative therapies with herbal/natural products. In the present study, we evaluated a well-characterized extract from the medicinal mushroom *Ganoderma lucidum* (GLE) for its affects on tumor growth and breast-to-lung cancer metastasis. MDA-MB-231 human breast cancer cells were implanted into the mammary fat pads of nude mice. GLE (100 mg/kg/every other day) was administered to the mice by an oral gavage for 4 weeks, and tumor size was measured using microcalipers. Lung metastases were evaluated by hematoxylin and eosin (H&E) staining. Gene expression in MDA-MB-231 cells was determined by DNA microarray analysis and confirmed by quantitative PCR. Identified genes were silenced by siRNA, and cell migration was determined in Boyden chambers and by wound-healing assay. Although an oral administration of GLE only slightly suppressed the growth of large tumors, the same treatment significantly inhibited the number of breast-to-lung cancer metastases. GLE also downregulated the expression of genes associated with invasive behavior (*HRAS*, *VIL2*, *S100A4*, *MCAM*, *I2PP2A* and *FN1*) in MDA-MB-231 cells. Gene silencing of *HRAS*, *VIL2*, *S100A4*, *I2PP2A* and *FN1* by siRNA suppressed migration of MDA-MB-231 cells. Our study suggests that an oral administration of GLE can inhibit breast-to-lung cancer metastases through the downregulation of genes responsible for cell invasiveness. The anti-metastatic benefits of GLE warrant further clinical studies.

## Introduction

Breast cancer is a leading cause of cancer death in women worldwide and the second leading cause of cancer death in the United States (1,2). The high mortality among cancer patients is associated with cancer metastasis, which contributes to more than 90% of cancer-related fatalities (3). Although chemotherapy, radiation therapy and targeted therapy can directly kill cancer cells, some cancer cells are resistant to these treatments and can further proliferate and metastasize. Therefore, identifying new drugs/compounds with an anti-invasive potential would help to control the metastatic properties of cancer cells. Interestingly, some natural/dietary compounds show the potential to suppress proliferation and invasiveness of cancer cells (4).

One of the dietary compounds not widely consumed in the United States is the mushroom. However, two recent epidemiological studies from Asia suggest that mushrooms can actually protect against breast cancer (5,6). *Ganoderma lucidum* is a mushroom recognized by traditional Chinese medicine (TCM) and commonly used in the forms of tea, powder and dietary supplements (7). The botanical characterization, description and therapeutic effects of *G. lucidum* are summarized in the American Herbal Pharmacopoeia (Reishi Mushroom; www.herbal-ahp.org). We have previously shown that *G. lucidum* extract (GLE) containing triterpenes and polysaccharides, suppresses the invasive behavior of breast cancer cells (8,9). Experimental *in vivo* studies demonstrated the inhibition of liver and lung metastases of lung carcinoma cells by triterpenoid fraction of *G. lucidum* and isolated ganoderic acid Me (GA-Me) and T (GA-T), respectively (10–12). In addition, 2.5% of the antlered form of *G. lucidum* in the diet suppressed the number of lung metastases of lung cancer cells (13). Oral administration of a lucidenic acid-rich *G. lucidum* extract inhibited lung and liver metastases of human hepatoma cells in a xenograft model (14).

In the present study, we evaluated the effect of GLE on the growth and breast-to-lung cancer metastasis in an orthotopic xenograft model without significantly influencing primary tumor growth. Our data suggest that an oral application of GLE inhibits lung metastases and can be used for the natural/alternative therapy of invasive breast cancers.

## Materials and methods

### Materials

Human breast cancer cells (MDA-MB-231) were obtained from ATCC (Manassas, VA). MDA-MB-231 cells were maintained in DMEM medium supplemented with penicillin (50 U/ml), streptomycin (50 U/ml), and 10% fetal bovine serum (FBS). Media and supplements were from Invitrogen (Grand Island, NY). FBS was obtained from Hyclone (Logan, UT). GLE was supplied by Pharmanex (Provo, UT). GLE is a standardized *Ganoderma lucidum* extract containing 6% triterpenes and 13.5% polysaccharides; the extraction procedure was previously described (15). GLE stock solution was prepared in water for animal experiments or in DMSO for cell culture experiments. siRNA reagents, scrambled siRNA and siRNA for *HRAS*, *VIL2*, *S100A4*, *MCAM*, *I2PP2A*, and *FN1* were from Santa Cruz Biotechnology (Santa Cruz, CA).

### Human breast tumor xenograft experiments

MDA-MB-231 cells (1×10^6^) in 0.2 ml DMEM were injected into the mammary fat pad of 6- to 7-week-old female nude mice (Harlan, Indianapolis, IN), as previously described (16). Three to four weeks after implantation with tumor cells, when tumors reached approximately 600 mm^3^, the animals were randomized into control and treatment groups (18 animals per group). The animals received intragastrical gavage every other day with water (control) or 100 mg GLE/kg of body weight (treatment) for an additional 28 days. The tumor size was measured using calipers, and the tumor volume was estimated by the formula: tumor volume (mm^3^) = W^2^ × L × 1/2, where L is the length and W is the width of the tumor. At the end of the experiment (day 28), the lungs were harvested and fixed in 10% neutral buffered formalin at 4°C for 24 h. Tissue was then processed overnight and embedded in paraffin. Five-micrometer sections were stained with hematoxylin and eosin (H&E), and the metastases in whole sections of stained lungs from 6 animals in each of the control and GLE-treatment groups were evaluated under a light microscope by 3 independent observers. The protocol for animal experiments was approved by the Animal Research Committee at IU Health Methodist Hospital according to the NIH guidelines for the Care and Use of Laboratory Animals.

### DNA microarrays

MDA-MB-231 cells were treated with GLE (0 and 1.0 mg/ml) for 24 h and total RNA was isolated with RNAeasy (Qiagen, Valencia, CA). This RNA was used for the evaluation of gene expression with Oligo GEArray Human Tumor Metastasis Microarray according to the manufacturer’s protocol (SA Biosciences, Frederick, MD, USA).

### Quantitative RT-PCR

The quantitative real-time polymerase chain reaction (qRT-PCR) was performed using the ABI Prism 7900HT Fast Real-Time PCR System (Applied Biosystems, Foster City, CA) according to the manufacturer’s instructions. MDA-MB-231 cells were treated with GLE (0 and 1.0 mg/ml) for 24 h and total RNA was isolated using RNAeasy (Qiagen). The RNA samples were reverse transcribed into cDNA (RT-PCR) using random hexamer primers and the TaqMan reverse transcription kit (Applied Biosystems). The cDNA (100 ng per sample) was subjected to qPCR analysis in quadruplicate using forward and reverse primers, the TaqMan Universal Master Mix, and a probe (10 *μ*l per reaction) in fast optical 96-well plates. The data were analyzed using the ABI Prism 7900 relative quantification (ΔΔCt) study software (Applied Biosystems). We used primers for *HRAS*, *VIL2*, *S100A4*, *MCAM*, *I2PP2A* and *FN1* genes with the *β-actin* gene as the internal control (Applied Biosystems). The gene expressions levels were normalized to β-actin and are presented as arbitrary fold changes compared between the control and GLE-treated cells.

### siRNA experiments

MDA-MB-231 cells (2×10^5^) were seeded into 6-well plates and incubated at 37°C in a 5% CO_2_ incubator until 70–80% confluent. The cells were transfected with control RNA (scrambled, scRNA) or siRNA according to the manufacturer’s protocol (Santa Cruz Biotechnology). Gene silencing by siRNA in MDA-MB-231 cells was evaluated by western blot analysis.

### Western blot analysis

MDA-MB-231 cells were treated with GLE (0 and 1.0 mg/ml) for 24 h. Whole cell extracts were isolated as described (15), membrane extracts were isolated by using a ProteoExtract^®^ subcellular proteome extraction kit (Merck, Darmstadt, Germany) according to the manufacturer’s protocol. Protein expression was detected by western blot analysis with the corresponding antibodies anti-HRAS, anti-ezrin, anti-S100A4, anti-MCAM, anti-SET, anti-fibronectin, and anti-β-actin, anti-GAPDH, and anti-α-integrin 3 as loading controls (Santa Cruz Biotechnology) as previously described (15). Reactive bands were visualized with a respective secondary antibody via an enhanced chemiluminescence (ECL) detection system.

### Cell migration assay

The effect of gene silencing on cell migration of MDA-MB-231 cells was assessed in Boyden chambers as previously described (17). After fixing and staining, the number of migrating cells was counted from at least four random fields using a microscope at ×20 magnification (17). Data points represent the average SD of individual filters within one representative experiment repeated at least twice.

### Wound healing assay

MDA-MB-231 cells were untransfected (control) or transfected with scRNA or siRNAs. After 24 h the cells were scratched using a 200-*μ*l pipette tip and further incubated for an additional 24 h. The extent of wound healing was observed microscopically and recorded.

### Statistical analysis

Data are represented as mean ± SD and were analyzed using SigmaPlot 11.2 (Systat Software Inc, San Jose, CA, USA).

## Results and Discussion

**Figure 1.**
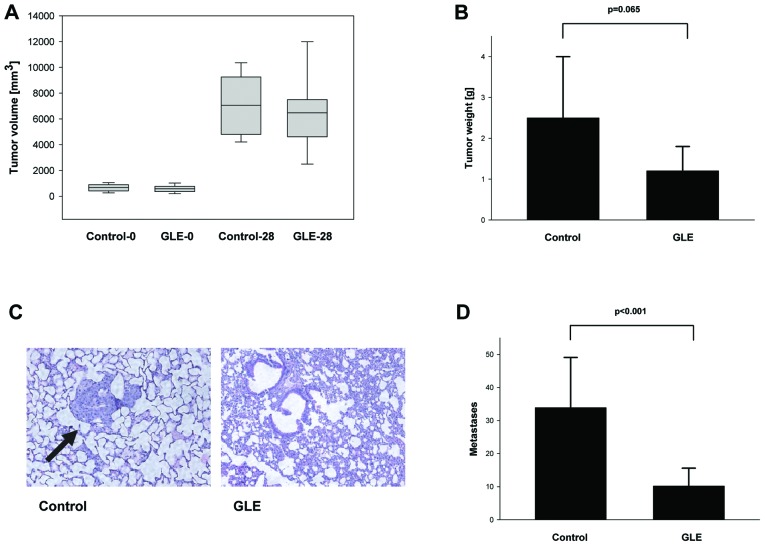
Effect of GLE on tumor growth and breast-to-lung cancer metastases. MDA-MB-231 breast cancer cells were implanted in the mammary fat pads of nude mice and treated with GLE as described in Materials and methods. (A) The size and (B) weight of tumors were measured after 28 days (n=16–18). (C) Representative H&E stained lungs from control and GLE-treated groups; black arrows indicate metastasis. (D) Lung metastases were quantified as described in Materials and methods (P<0.001, n=6).

Although previous studies demonstrated the suppression of tumor growth and the inhibition of metastases by purified *Ganoderma lucidum* compounds or extracts in experimental animals, these studies usually started the treatment with small tumors close to 100 mm^3^ in size (12,14). Since not all breast cancers are diagnosed in the early stages, we were interested to learn whether GLE inhibits the growth and metastases of larger tumors. Highly invasive human breast cancer cells MDA-MB-231 were injected into the mammary fat pads of mice, and an oral application of GLE (100 mg/kg/body weight every other day) was started when the tumors reached 600 mm^3^. GLE treatment for 4 weeks had modest inhibitory effects on tumor size and weight (Fig. 1). Since we have previously shown that GLE inhibits invasive behavior in MCF-7 and MDA-MB-231 breast cancer cells *in vitro* (8,9), we further studied whether GLE inhibits breast-to-lung cancer metastases *in vivo*. Although we did not observe changes in the tumor volumes in the control and GLE-treatment groups in MDA-MB-231 cell-derived tumors (Fig. 1A), we found statistically non-significant inhibition of tumor growth by GLE (Fig. 1B). This effect was caused by the necrosis since both control and GLE-treated tumors had necrotic central regions that were filled with fluid. However, our data show significant inhibition of breast-to-lung cancer metastases from 33.9±15.2 in control to 10.2±5.4 in GLE-treated animals (P<0.001) (Fig. 1C and D).

**Figure 2.**
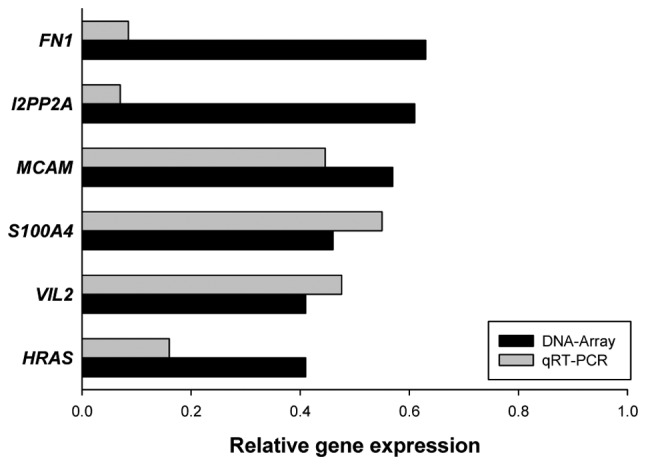
Effect of GLE on human tumor metastasis genes. MDA-MB-231 cells were treated with vehicle or GLE for 24 h and the gene expression was determined by Oligo GEArray Human Tumor Metastasis Microarray and confirmed by qRT-PCR as described in Materials and methods. The data are averages from 2 (microarray) and 4 (qRT-PCR) experiments. The fold change is relative to vehicle-treated cells.

In order to identify which pro-metastatic genes are affected in MDA-MB-231 cells by GLE, MDA-MB-231 cells were treated with vehicle or GLE (24 h, 1.0 mg/ml) and gene expression was analyzed by Oligo GEArray Human Tumor Metastasis Microarray as described in Materials and methods. GLE treatment downregulated the expression of *HRAS*, *VIL2*, *S100A4*, *MCAM*, *I2PP2A* and *FN1* genes by more than 20%, which we further confirmed by qRT-PCR (Fig. 2).

**Figure 3.**
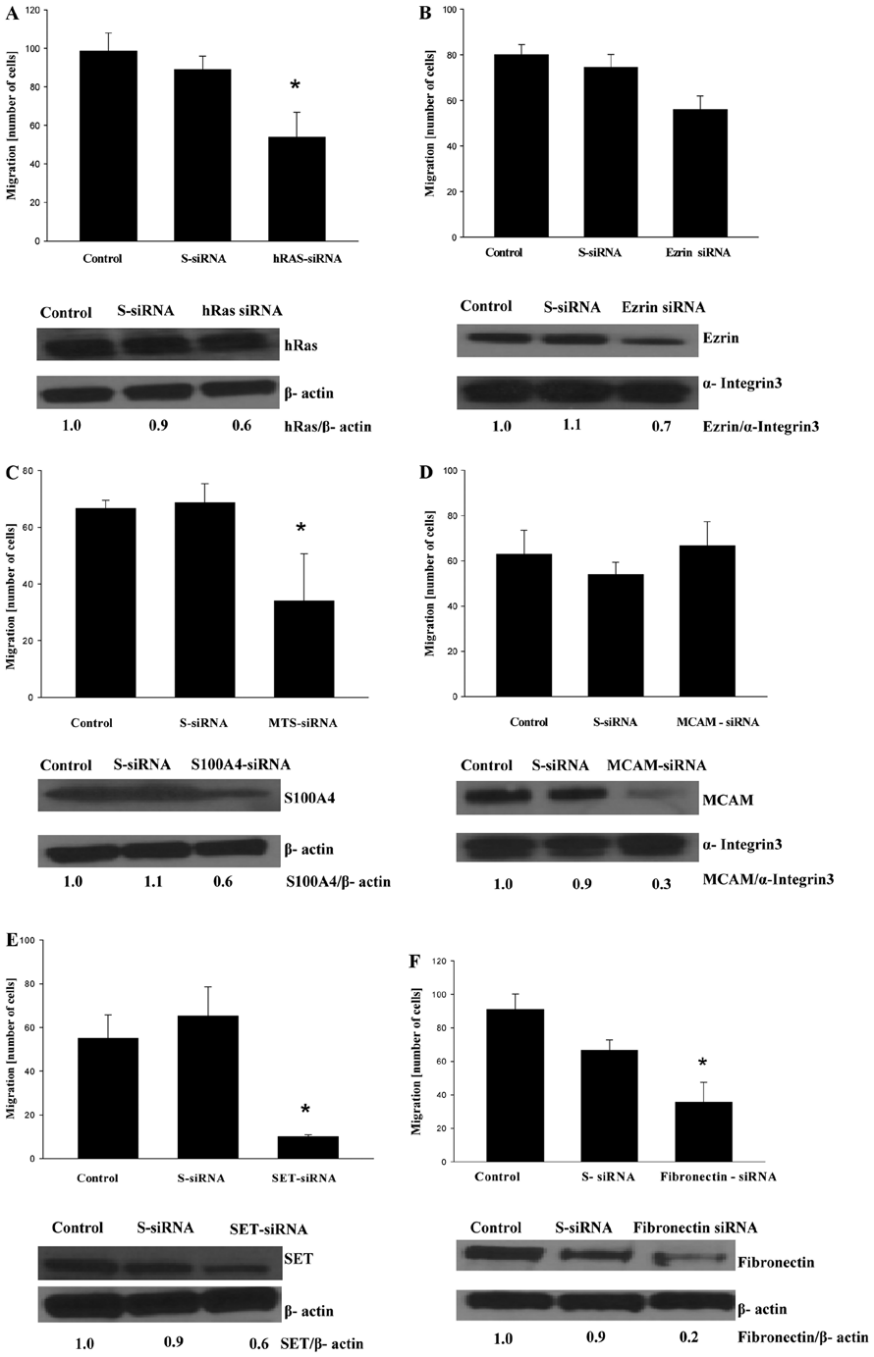
Effect of genetic silencing of GLE downregulated genes on cell migration. MDA-MB-231 cells were untransfected (control) or transfected with scrambled (sc-siRNA) or specific gene siRNA and cell migration and proper gene silencing was evaluated as described in Materials and methods. (A) HRAS, (B) VIL2 (ezrin), (C) S100A4, (D) MCAM, (E) I2PP2A (SET), (F) FN1 (fibronectin). The data are mean ± SD (n=3), P≤0.05 by ANOVA. Western blots show representative experiment.

**Figure 4.**
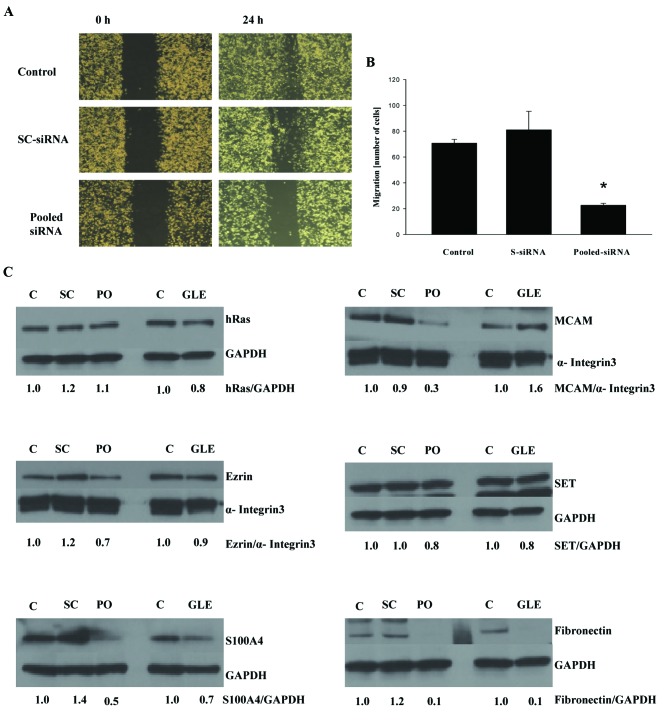
Pooled siRNA inhibits cell migration. MDA-MB-231 cells were untransfected (control) or transfected with scrambled (sc-siRNA) or a pool of siRNAs, (pooled-siRNA, containing HRAS-siRNA, VIL2-siRNA, S100A4-siRNA, MCAM-siRNA, I2PP2A-siRNA, and FN1-siRNA). Cell migration was evaluated by (A) the wound healing assay and (B) cell migration assay described in Materials and methods. The data are mean ± SD (n=3), p≤0.05 by ANOVA. (C) Representative western blots from MDA-MB-231 cells transfected with scrambled siRNA (SC) or pooled-siRNA (PO), or treated with GLE (GLE) or control (C).

To confirm that genes targeted by GLE are indeed responsible for the invasiveness of MDA-MB-231 cells, we silenced these genes by siRNA to evaluate whether this genetic manipulation suppressed the migration of MDA-MB-231 cells. Increased expression of the oncogene *HRAS* is associated with aggressive breast cancer, and an overexpression of *HRAS* induces cell migration and an invasive phenotype in breast epithelial cells (18). Not surprisingly, *HRAS* silencing suppressed the migration of MDA-MB-231 cells (Fig. 3A). In agreement with a previous study by Li *et al* (19) on the silencing of *VIL2*, coding ezrin (VIL2), a cytoplasmic peripheral membrane protein that plays a key role in cell motility, slightly suppressed the migration of MDA-MB-231 cells (Fig. 3B). The S100A4 protein is overexpressed in highly metastatic cancers and controls cell migration through different pathways (20). Gene silencing of *S100A4* also suppressed the migration of MDA-MB-231 cells (Fig. 3C). At the time of our manuscript preparation, Wang *et al* (21) demonstrated that gene silencing of *S100A4* inhibited cell migration and invasion as well as lung metastases of MDA-MB-231 cells in mice. Although the original studies suggested that *MCAM* (also known as CD146 membrane glycoprotein) is a tumor suppressor in breast carcinomas (22), CD146 expression was later associated with a poor prognosis in breast cancer patients and increased motility of breast cancer cells (23,24). However, gene silencing of *MCAM* did not affect the migration of MDA-MB-231 cells (Fig. 3D). The SET protein (gene *I2PP2A*) inhibits protein phosphatase 2A (PP2A), which regulates oncoproteins (e.g. c-Myc, Bcr-Abl) in various cancers (25,26). Therefore, the inhibition of *I2PP2A* is therapeutically important, and, as recently demonstrated, targeting SET suppresses lung tumors (27). As seen in Fig. 3E, gene silencing of *I2PP2A* also suppressed SET protein expression and the inhibited migration of MDA-MB-231 cells. The fibronectin 1 protein (gene *FN1*) controls cell adhesion and migration, and FN1 overexpression was detected in breast cancer metastases (28,29). Gene silencing of *FN1* resulted in the downregulation of FN1 expression and inhibited the migration of MDA-MB-231 cells (Fig. 3F). Since cell migration is a complex process and is controlled by more than one protein, we evaluated whether the gene silencing of all genes whose expression was downregulated by GLE treatment inhibited cell migration to a larger extent than the silencing of these genes individually. MDA-MB-231 cells were transfected with a mixture of siRNAs for *HRAS*, *VIL2*, *S100A4*, *MCAM*, *I2PP2A* and *FN1*, and cell migration was evaluated. As seen in Fig. 4A, transfection of pooled siRNAs suppressed migration in the wound-healing assay as well as in the cell migration assay in Boyden chambers (Fig. 4B), suggesting that targeting a pool of pro-invasive genes is a better strategy than targeting only one gene. On the other hand, the expression of ezrin, S100A4, MCAM, SET and fibronectin was downregulated by pooled siRNA, whereas the expression of HRAS was not affected (Fig. 4C). In addition, pooled siRNA and GLE treatment demonstrated the strongest inhibition of fibronectin expression, suggesting that fibronectin is the major target for inhibiting cell invasiveness. Although our data with pooled siRNA generally confirms our previous results with the isolated siRNA (Fig. 3), it is possible that pooled siRNA could also inhibit some of the single siRNA since HRAS-siRNA downregulated expression of the HRAS protein, whereas pooled siRNA does not. In addition, GLE treatment suppressed expression of different pro-invasive proteins with different potency, further suggesting specific targeting at transcriptional or posttranslational levels. These questions will be addressed in our future studies.

In our present study, we found that GLE downregulates the expression of a set of genes (*HRAS*, *VIL2*, *S100A4*, *MCAM*, *I2PP2A* and *FN1*) that are different from the previously published genes that mediate breast-to-lung cancer metastasis (30). One of the reasons for this difference is that in our experiments we originally evaluated the effect of GLE on the expression of selected pro-metastatic genes by using Oligo GEArray, which does not cover all genes. Moreover, the breast-to-lung metastatic genes identified by Minn *et al* (30) were overexpressed in the lung-metastatic derivative of MDA-MB-231 but not in the parental MDA-MB-231 cells used in our study. Therefore, a different set of genes should be targeted during the progression of metastatic breast cancer. In addition, our study was performed with only one cell line of highly metastatic triple negative breast cancer cells, MDA-MB-231 and different genes can be targeted in other metastatic breast cancers.

In conclusion, the chemically characterized dietary mushroom extract GLE inhibits breast-to-lung cancer metastasis of highly invasive human breast cancer cells implanted in mouse mammary tissue. In addition, GLE suppresses the expression of genes involved in the invasive behavior of cancer cells. Further preclinical studies evaluating GLE activity in the prevention of breast cancer metastasis are warranted.

## Abbreviations:

GLE: *Ganoderma lucidum* extract
VIL2: ezrin
FN1: fibronectin 1
MCAM: melanoma cell adhesion molecule
S100A4: S100 calcium binding protein A4
I2PP2A: SET nuclear oncogene
HRAS: v-Ha-Ras Harvey rat sarcoma viral oncogene homolog
